# Prevalence of depressive symptoms and its burden on neurological practice in urban Egypt: a cross-sectional study

**DOI:** 10.1038/s41598-026-44875-w

**Published:** 2026-04-11

**Authors:** Maram Samy Nasef, Sara Abozeid, Saifeldin A. Hassan

**Affiliations:** 1 Neurology department, Faculty of Medicine, New Giza University, Giza, Egypt; 2 Faculty of Medicine, New Giza University, Giza, Egypt

**Keywords:** Depression, Neurologists, Diseases, Health care, Neurology, Neuroscience

## Abstract

Neurologists, as specialists in a high-stress field, which hold lots of mental and emotional stressors. The complexity of neurological conditions, the extended working hours, and the emotional burden of managing chronic or terminal patients contributes to a stressful work environment. Globally, studies have shown that healthcare professionals, particularly those in specialties of high demand like neurology, are at risk of depression and burnout. This study aims to assess the prevalence of depressive symptoms among neurologists in Egypt. Help identify the risk factors that contribute to these depressive symptoms. A cross-sectional survey was conducted among Egyptian neurologists working in both public and private healthcare institutions. Participants in the study completed the Patient Health Questionnaire-9 (PHQ-9) to assess the severity of depressive symptoms. Data on demographics, job satisfaction, shift duration, workload and overall job satisfaction were also collected and analyzed using descriptive and inferential statistics. Out of 138 neurologists surveyed, nearly half (43.5%) reported moderate to severe depressive symptoms. Those with a prior diagnosis of depression were especially affected—more than 8 in 10 (82.4%, 95% CI 66.2%–91.7%) reported significant symptoms, compared to just over a third (37.5%, 95% CI 28.8%–47.1%) of those without a known history (*p* < 0.001). Higher depression scores were also linked to early career stage and longer working hours. Over half of the neurologists with less than three years of experience (53.1%) and those working more than 80 h per week (56.3%) screened positive for moderate to severe depression. These findings highlight a high burden of depressive symptoms among Egyptian neurologists, particularly those early in their careers or with a prior history of depression, emphasizing the urgent need for targeted mental health interventions and systemic workplace reforms.

## Introduction

Depression among healthcare professionals is a growing concern with studies consistently showing high rates of depressive symptoms across medical specialties. While much of the literature has focused on GPs, residents and surgical specialties, the mental health of neurologists has been significantly understudied. Neurologists, as specialists in a high-stress field, which hold lots of mental and emotional stressors. The complexity of neurological conditions, the extended working hours, and the emotional burden of managing chronic or terminal patients contributes to a stressful work environment.

From a biological perspective, depression is associated with dysregulation of multiple neurobiological systems, including the hypothalamic–pituitary–adrenal (HPA) axis, monoaminergic neurotransmission, neuroinflammation, and impaired neuroplasticity^1^. Chronic occupational stress, common among physicians, leads to sustained cortisol secretion and HPA axis hyperactivity, which has been linked to hippocampal atrophy, cognitive impairment, and mood dysregulation^2^. Additionally, prolonged stress and sleep deprivation frequent in medical practice are associated with alterations in serotonin and dopamine pathways, increased pro-inflammatory cytokines, and reduced brain-derived neurotrophic factor (BDNF), all of which contribute to the pathophysiology of depression^1,3,4^. These biological mechanisms may be particularly relevant in physicians, whose repeated exposure to stress, high cognitive load, and circadian disruption can amplify vulnerability to depressive disorders.

Globally, studies have shown that healthcare professionals, particularly those in specialties of high demand like neurology, are at risk of depression and burnout^5^. A meta-analysis by Mata et al. (2015) found 28.8% of resident physicians experience depressive symptoms, with some studies reporting rates as high as 43.2% depending on the screening tool used^6^. Most notably, a cross-sectional study conducted Indonesia by Perwitasari et al., in directly assessed depression among neurologists. In a national sample of 151 Indonesian neurologists, 36.4% had mild-to-moderate depression and 8.6% had severe symptoms as measured by the Beck Depression Inventory (BDI-II)^7^.

The study also found strong correlations between depressive symptoms and work related stressors such as long working hours, night shifts and higher workload in Egypt the health care system is under a large amount of pressure, with a shortage of mental health resources, huge number of patients and long shifts^8^ These factors end up increasing the mental health burden on neurologists, leading to psychological distress, burnout, and depression^8^. Despite the rising awareness of mental health issues among healthcare professionals, there is limited research on the prevalence and contributing risk factors of depression among neurologists in Egypt. Understanding the mental health status is crucial, as depression not only affects their overall well-being but also the quality of care they provide for their patients^9^. As far as the research known, there has been no study on depression conducted among neurologists in Egypt.

## Material and methods

The study was conducted in hospitals across Cairo and Giza governorates that have active neurology departments, including university hospitals, private hospitals, public/ insurance hospitals, and specialized neurology centers. The target population included practicing neurologists at different career stages (residents, registrars, specialists, and consultants) who were actively working in the selected hospitals from the first of April 2025 to first of May 2025. Participants needed to have worked a minimum of six-month experience in neurology, are currently working in one of selected hospitals and willing to provide informed consent. Neurologists on extended leave, unavailable during time of study or declined participation were excluded.

### Sampling technique

A two-stage cluster sampling technique was utilized to select the study sample.

#### Stage 1: cluster selection

Hospitals served as the primary sampling units (clusters). From a comprehensive list of hospitals with neurology departments in Cairo and Giza, hospitals were stratified by type: university hospitals, private hospitals, public/insurance hospitals, and specialized neurology centers. From each stratum, hospitals were randomly selected using simple random sampling, resulting in a total of eight hospitals (2 university, 4 private, and 2 public/insurance hospitals).

#### Stage 2: participant selection

Within each selected hospital, all eligible neurologists were invited to participate. In hospitals with a large number of neurologists, simple random sampling was applied to select a maximum of 4–5 participants to ensure representativeness and feasibility.

### Sample size

The sample size was calculated using the formula for estimating a proportion in a finite population, assuming a 30% prevalence of depression based on previous literature, a 95% confidence interval, and a 5% margin of error. To account for the cluster sampling design, a design effect of 1.5 was incorporated, yielding a minimum required sample size of approximately 120 neurologists.

### Data collection tools

Data was collected via a structured self-administered questionnaire on google forms that included demographic and occupational characteristics, The Patient Health Questionnaire-9 (PHQ-9) to assess the presence and severity of depression and additional questions related to workload, work-life balance, and perceived stress. The collected data was entered into the research data register for further analysis.

### Statistical analysis

The collected data were coded, tabulated, and statistically analyzed using IBM SPSS Statistics (Statistical Package for Social Sciences) software version 22.0 (IBM Corp., Chicago, USA, 2013). Categorical and nominal data were analyzed using the chi-square test, with Fisher’s exact test used when chi-square assumptions were not met. Quantitative data were presented as mean ± standard deviation (SD) for normally distributed variables, while qualitative data were presented as frequencies and percentages.

One-way analysis of variance (ANOVA) was used to assess differences in mean PHQ-9 scores and the prevalence of depression across different job categories. Linear regression analysis was performed to evaluate the relationship between continuous variables, including work hours and depression scores, as well as to assess trends in mean PHQ-9 scores across job titles. A p-value < 0.05 was considered statistically significant.

Univariate Analysis was performed to examine each variable individually. Variables significant in univariate analysis or clinically relevant were included in a multivariate logistic regression to calculate adjusted odds ratios with 95% confidence intervals.

## Results

### Demographic characteristics

138 neurologists participated in the study, they were asked demographic data, years of experience, job title, working hours, PHQ9 questionnaire and what do they believe would improve their overall job satisfaction. Most of the respondents were female (n = 89, 64.5%), while male neurologists accounted for (n-49, 35.5%). Most participants were aged 31–40 years (55.8%), followed by 20–30 years (35.5%), > 50 years (8.0%), and 41–50 years (0.7%). In terms of job title, the largest group of participants were residents (44.9%), followed by registrars (23.2%), consultants (15.2%), others (10.1%), and professors (6.5%). Years in practice showed that nearly 35% had < 3 years of experience, while 28.3% had > 10 years, and the remainder were spread across intermediate experience levels. Most neurologists reported working 36–72 h per week (48.6%), while 37% worked > 72 h, and 14.5% worked 24–36 h (Table 1).

**Table 1:** Demographic and occupational characteristics of the neurologists included in the study (n = 138).

Category	Subcategory	n	Percent
Gender	Female	89	64.5%
	Male	49	35.5%
Age group (years)	20–30	49	35.5%
	31–40	77	55.8%
	41–50	1	0.7%
	> 50	11	8.0%
Job title	Resident	62	44.9%
	Registrar	32	23.2%
	Consultant	21	15.2%
	Professor	9	6.5%
	Other	14	10.1%
Years of experience	< 3 years	48	~ 34.8%
	Intermediate levels	51	~ 37.0%
	> 10 years	39	28.3%
Working hours/Week	24–36 h	20	14.5%
	36–72 h	67	48.6%
	> 72 h	51	37.0%

As shown in Table 1, the majority of respondents were female and aged between 31 and 40 years. Most participants were in residency training, with varied levels of experience and work hours. The table also reflects a distribution of working hours, showing that nearly half of the neurologists worked between 36 and 72 h per week. This data provides context for interpreting PHQ-9 scores and participants’ views on factors that could improve job satisfaction.

### Depression prevalence

**Fig. 1:**
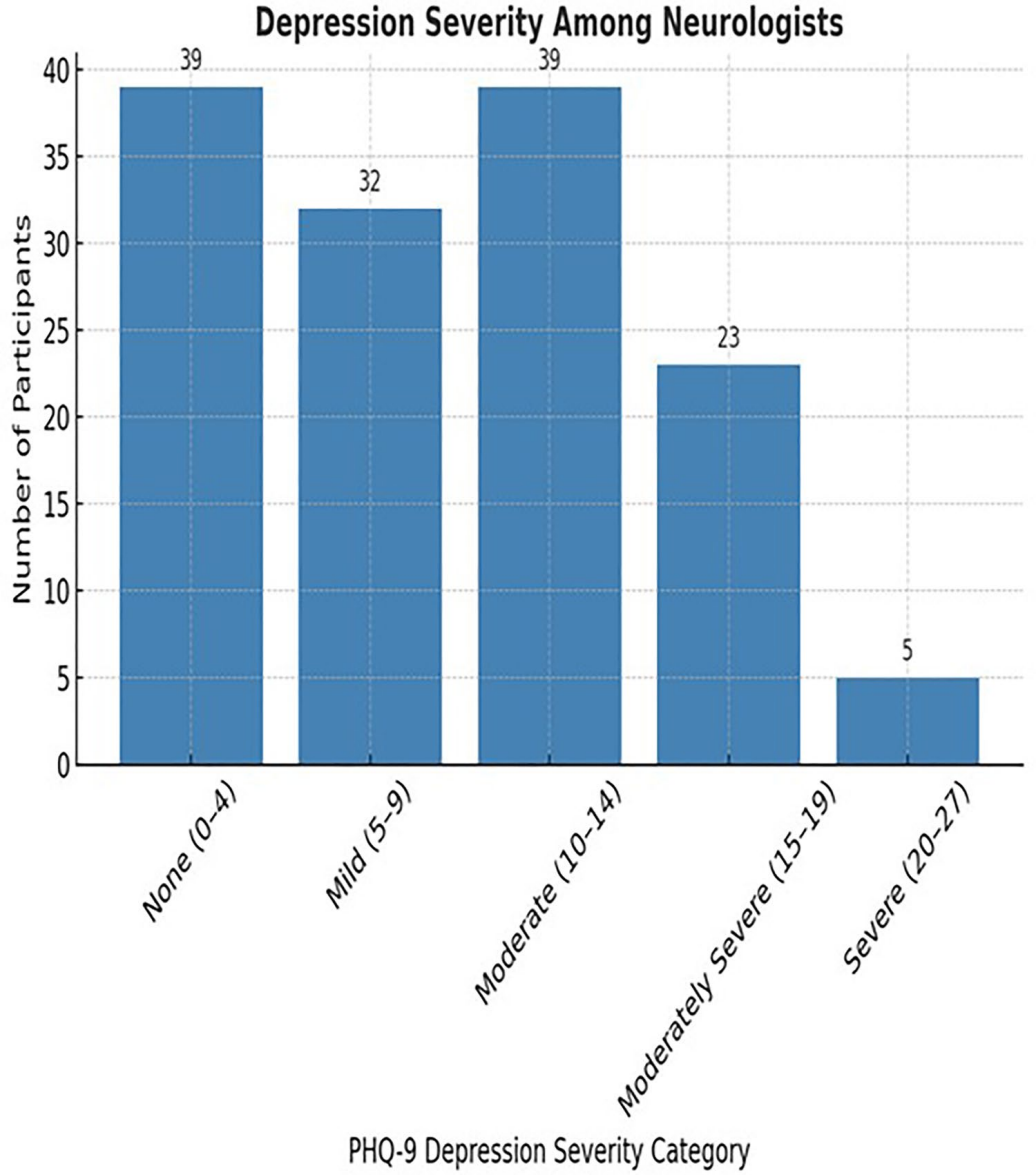
Depression severity among neurologists. This bar chart illustrates the distribution of PHQ-9 depression severity categories among neurologists. Most participants fall into the “None (0–4)” and “Moderate (10–14)” categories (each with 39 participants), followed by “Mild (5–9)” with 32 participants. Fewer participants reported “Moderately Severe (15–19)” (23 participants) and “Severe (20–27)” depression (5 participants), indicating a wide range of depression levels within the cohort.

As shown in Fig. 1 based on PHQ-9 scoring, a significant number of neurologists exhibited depressive symptoms of varying severity. Specifically, 32.6% of participants reported minimal or no depression (PHQ-9 score 0–4; 95% CI 25.2%–40.8%), while 34.8% experienced mild depression (score 5–9; 95% CI 27.3%–43.0%). Additionally, 20.3% met criteria for moderate depression (score 10–14; 95% CI 14.3%–27.9%), and 12.3% scored in the moderately severe to severe range (score ≥ 15; 95% CI 7.7%–18.9%).

### Predictors of depression

The participants were asked about the number of years in practicing neurology and results showed that there was a significant decrease in depression severity with increasing years of practice. Analysis of PHQ-9 scores across age groups revealed no statistically significant association between age and depression severity among neurologists. The correlation between age and total PHQ-9 score was weak and negative (*r* = *-0.05*), indicating that depression levels remained relatively consistent across different age categories. Among neurologists with < 3 years of experience**,** 53.1% reported moderate to severe depressive symptoms, whereas this proportion dropped to 19.4% among those with > 10 years of experience.

Even though the association between experience and depression did not reach conventional levels of statistical significance (*p* = *0.06*), the trend suggests a clinically relevant pattern. Confidence intervals for moderate to severe depression were widest in the least experienced group (*95% CI 39.4%–66.4%*), reflecting both a high burden and variability in reported symptoms.

**Fig. 2:**
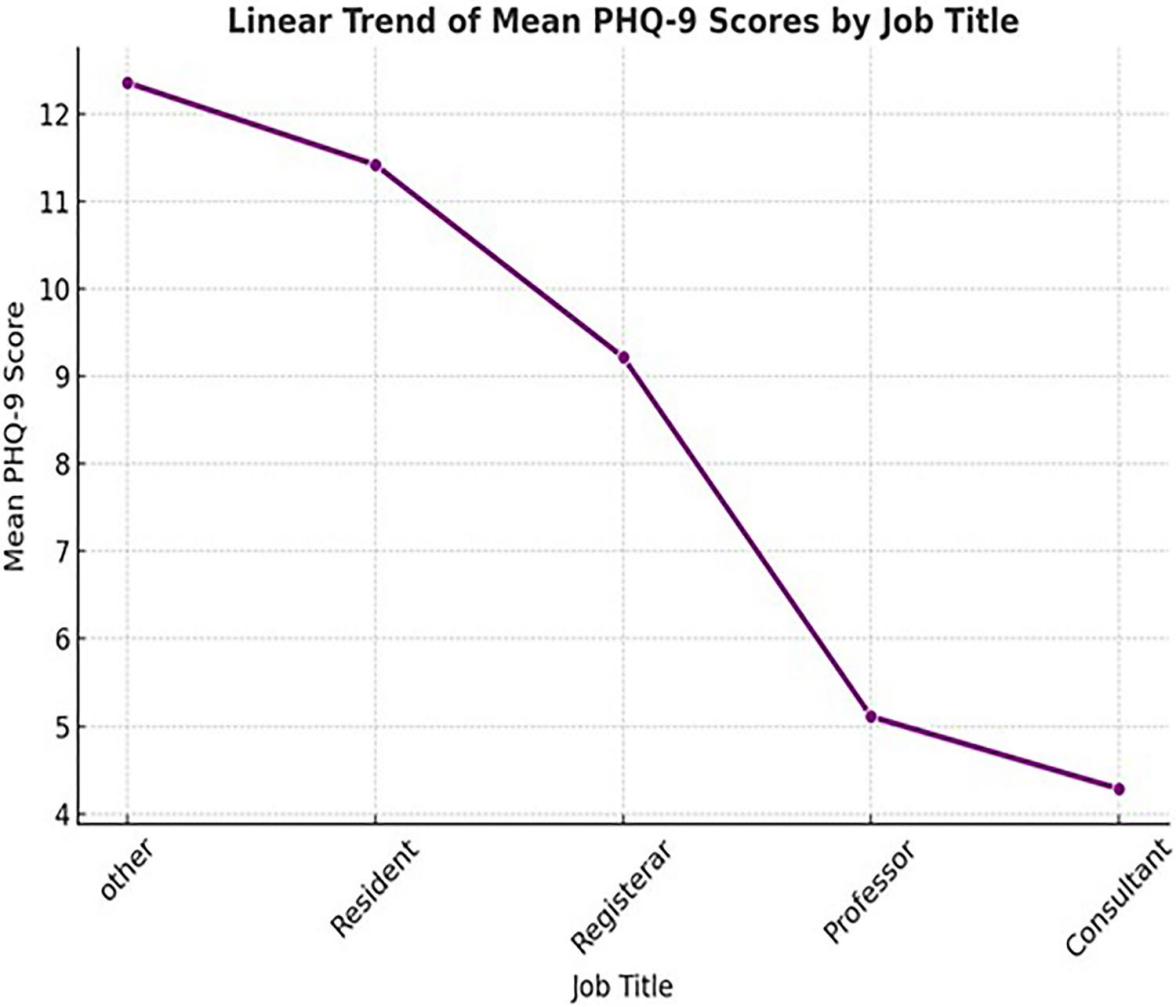
Trend of mean PHQ-9 scores by job title. As shown in the figure the line graph displays the average PHQ-9 depression scores across different job titles. Individuals in the “Other” category reported the highest mean scores, followed by Residents and Registrars. Professors and Consultants had the lowest mean depression scores, indicating a possible inverse relationship between job seniority and depression severity.

As shown in Fig. 2 a significant association was found between job title and depression severity as measured by the PHQ-9 total score (*p* < 0.001). The mean PHQ-9 scores varied notably across job categories. Residents reported the highest average score of 11.4(SD = 6.3), followed closely by those classified as “Other” (12.4, SD = 3.0) and Registrars 9.2, (SD = 6.0). In contrast, Consultants and Professors reported significantly lower scores, averaging 4.3 (SD = 1.7) and 5.1 (SD = 1.1) respectively. These findings suggest that junior clinicians, particularly Residents and Registrars, are more likely to experience elevated depressive symptoms compared to their senior counterparts.

The differences were statistically significant based on one-way ANOVA (F = 6.79, *p* < 0.001), indicating that job title is a meaningful predictor of depression severity among neurologists.

**Fig. 3:**
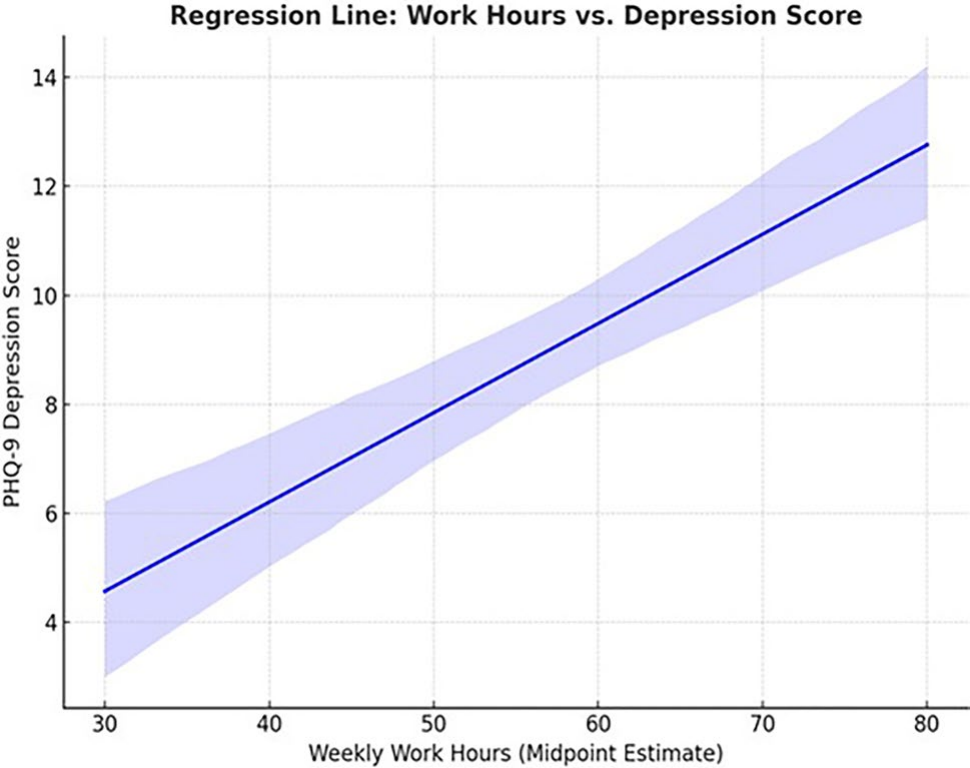
Regression line – work hours vs. depression score. As shown in the figure the scatter plot with a regression line shows a positive linear relationship between weekly work hours and PHQ-9 depression scores. As work hours increase, depression scores also tend to rise, suggesting that longer working hours are associated with higher levels of depressive symptoms among neurologists.

As shown in Fig. 3 statistical analysis revealed a moderate and statistically significant positive correlation between weekly working hours and PHQ-9 depression scores among neurologists. The Pearson correlation coefficient was r = 0.48, (p-value < 0.000001), indicating a highly significant association. The 95% confidence interval (CI) for the correlation coefficient ranged from 0.34 to 0.60, suggesting a consistent positive relationship, indicating that longer work schedules were associated with depressive symptoms particularly those exceeding 72 h (Fig. 3).

A comparison of depression severity between neurologists with and without a prior history of depression revealed significant differences. Among those with a history of depression, 82.4% (n = 28) reported moderate to severe symptoms**,** while only 17.6% (n = 6) had none or mild symptoms. In contrast, among neurologists with no history of depression, just 37.5% (n = 39) reported moderate or worse symptoms, with 62.5% (n = 65) reporting none or mild symptoms. This difference was statistically significant (*p* < 0.001), indicating that a prior diagnosis of depression is strongly associated with a higher likelihood of currently experiencing moderate or severe depressive symptoms.

The neurologists who reported no prior diagnosis of depression (n = 104) who had symptoms of depression was notably high. It should be noted though that there were no participants who reported scores consistent with severe depression (PHQ-9 score ≥ 20), with a corresponding 95% CI ranging from 0.0 to 3.6%. Overall, 37.5% of neurologists in this subgroup exhibited moderate to moderately severe depression (PHQ-9 score ≥ 10; 95% CI 28.8%–47.1%), reflecting a considerable burden of clinically significant depressive symptoms in a population with no known prior diagnosis of mental health affection.

**Fig. 4:**
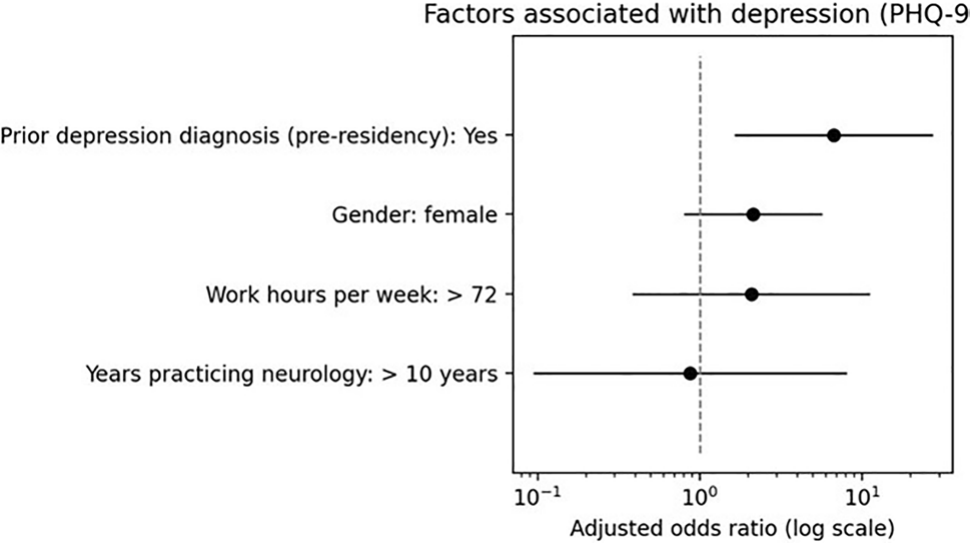
Adjusted odds ratio of predictors of depression. As shown in the figure Forest plot Displaying adjusted odds ratio for predictors of depression the X axis represents adjusted odds ratio with corresponding confidence intervals and the Y axis lists the predictors assessed including prior diagnosis of depression before residency, female gender, working more than 72 h per week and more than 10 years of practicing neurology. A prior diagnosis of depression demonstrated their strongest association with depression (aOR = 6.71) while having more than 10 years of neurology practice was associated with the lowest adjusted odds ratio.

In adjusted logistic regression analyses, a prior diagnosis of depression before residency was strongly associated with current depression (PHQ-9 ≥ 10), with substantially higher odds among those reporting a prior diagnosis (aOR 6.71, 95% CI 1.65–27.32; p≈0.008). Female gender was associated with higher odds of depression in the adjusted model (aOR 2.13, 95% CI 0.80–5.68), although this association did not reach statistical significance. Working longer hours showed an association in the expected direction, with higher odds observed among those working more than 72 h per week (aOR 2.08), but estimates were imprecise with wide confidence intervals (95% CI 0.38–11.22). Age group and years in practice were included as covariates, but their adjusted associations were smaller and less precise than that observed for prior depression (Fig. 4).

In univariate analyses, several factors showed notable associations with depression. Lack of career satisfaction was strongly associated with higher odds of depression (OR 6.92, 95% CI 3.27–14.62), as was a prior diagnosis of depression before residency (OR 7.78, 95% CI 2.96–20.46). Working more than 72 h per week was associated with increased odds in comparison with working 36–72 h (OR 2.79, 95% CI 1.31–5.95). Male gender was associated with lower odds of depression (OR 0.36, 95% CI 0.17–0.75), and longer duration of neurology practice (> 10 years) was protective relative to < 3 years (OR 0.37, 95% CI 0.15–0.88). Younger age (20–30 years) showed higher odds compared with 31–40 years, although this estimate was imprecise (OR 1.49, 95% CI 0.72–3.07). All associations are unadjusted and should be interpreted as bivariate (Table 2).

**Table 2:** Univariate analysis of predictors of depression.

Predictor (level vs reference)	n (level)	OR (95% CI)
Are you overall satisfied with your career?: No vs Yes	65	6.92 (3.27–14.62)
Gender: Male vs female	49	0.36 (0.17–0.75)
Have you been diagnosed with depression prior to your residency? Yes vs No	34	7.78 (2.96–20.46)
How many hours do you work per week?: > 72 vs (36 – 72)	51	2.79 (1.31–5.95)
Estimated number of years you have been practicing neurology?: > 10 years vs < 3 years	39	0.37 (0.15–0.88)
Age: (20 – 30) vs (31- 40)	49	1.49 (0.72–3.07)

**Table 3:** Multivariate linear regression model for predictors of depression.

Predictor	Reference	Level (vs reference)	n	aOR (95% CI)
Age (years)	20—30	> 50	11	0.42 (0.01–27.34)
Age (years)	20—30	41—50	1	0.88 (0.0–666.45)
Age (years)	20—30	31- 40	77	1.6 (0.08–30.26)
Career satisfaction	No	Yes	73	0.23 (0.09–0.6)
Gender	Male	Female	89	2.13 (0.8–5.68)
Prior depression diagnosis (preresidency)	No	Yes	34	6.71 (1.65–27.32)
Work hours per week	24—< 36	36—72	67	1.79 (0.51–6.37)
Work hours per week	24—< 36	> 72	51	2.08 (0.38–11.22)
Years practicing neurology	3- < 5 years	8—10 years	15	0.19 (0.01–3.19)
Years practicing neurology	3- < 5 years	5—8	30	0.3 (0.03–3.22)
Years practicing neurology	3- < 5 years	> 10 years	39	0.87 (0.09–8.02)
Years practicing neurology	3- < 5 years	< 3 years	48	1.79 (0.24–13.38)

As shown in the table, analysis of factors associated with depression. The table presents unadjusted odds ratio for depression according to demographic characteristics and work-related factors including gender, age overall reaction job satisfaction, prior diagnosis of depression, weekly work hours, years of practicing neurology. Odds ratio reflect the association of each variable with depression when analyzed independently.

In response to the question on improving job satisfaction, the most frequently reported factor was the need for a good training program and a clear career path, cited by 34.8% of respondents. The 95% confidence interval (CI) for this proportion ranged from 27.3 to 43.0%. This was followed by better salary (26.8%, 95% CI 20.1%–34.8%), less work hours (22.5%, 95% CI 16.3%–30.1%), and better work environment (15.9%, 95% CI 10.8%–23.0%). Although no formal hypothesis test (and thus no p-value) was conducted for comparing proportions in this case, the non-overlapping confidence intervals suggest meaningful differences in perceived priorities.

## Discussion

A cross-sectional study involving 138 neurologists in Egypt was conducted to evaluate the prevalence and severity of depressive symptoms. Results indicated that 32.6% of participants reported minimal or no depressive symptoms, 34.8% experienced mild depression, 20.3% met criteria for moderate depression, and 12.3% reported symptoms in the moderately severe to severe range. These findings reflect a higher prevalence of depression compared to a previous study, which reported a rate of 18.4% among neurologists^10^, although the observed rates fall within the range documented in studies involving physicians and surgeons more broadly (22.2%–37.8%)^6,11,12^, with the exception of one outlier study that reported a prevalence of 6.2%^13^.

The prevalence of depressive symptoms in our sample (67.4% with mild to severe symptoms) is higher than rates typically reported in general population studies. Although our study did not include a non-medical control group, comparison with published literature suggests that depression is substantially more prevalent among healthcare professionals. For example, Abdalgeleel et al. (2023) studied 1511 Egyptian healthcare workers, of whom physicians constituted 77.8% of the sample, and reported that approximately 96% of participants had mild to severe depressive symptoms. These findings are consistent with our results and support the notion that medical professionals experience a significantly elevated psychological burden compared to general population samples^14^.

### Age, gender, and depression

Age groups revealed no statistically significant association with depression severity among neurologists in the present study. The correlation between age and total PHQ-9 score was weak and negative (r = − 0.05), suggesting relatively stable depression levels across different age categories. This finding contrasts with previous reports indicating lower depression rates among older physicians^12,15^. A large-scale study conducted in 2022 by Chen et al.^12^, involving 15,243 emergency physicians and using the PHQ-9, found that older age was a protective factor, with physicians aged > 31 years being less likely to experience depression compared to younger counterparts. Similarly, Liang et al.^15^ reported that younger medical staff (≤ 30 years) demonstrated significantly higher depression scores, assessed using the Self-Rating Depression Scale (SDS), compared to those aged > 30 years.

The discrepancy between these findings and our results may be attributable to differences in study populations, clinical specialties, assessment tools, and healthcare system contexts. In addition, the relatively narrow age distribution within our neurologist cohort and the predominance of early-career physicians may have limited the ability to detect age-related differences in depression severity.

Regarding Gender, Females were associated with higher odds of depression in the adjusted model (aOR 2.13, 95% CI 0.80–5.68), although this association did not reach statistical significance. While in Univariate models, Male gender was associated with lower odds of depression (OR 0.36, 95% CI 0.17–0.75). Several studies support the observed trend of higher odds of depressive symptoms among female neurologists. A large systematic review and meta-analysis by Mata et al. (2015) demonstrated that female physicians, particularly residents, consistently reported higher prevalence of depression and depressive symptoms compared with their male counterparts^6^. Similarly, Shanafelt et al. (2012) found that female physicians experienced greater emotional exhaustion and poorer work–life balance, both of which are strongly associated with depressive symptomatology^9^. Neurology-specific studies conducted in Indonesia by Putri et al. (2021) and Perwitasari and Hidayat (2024) also reported higher levels of depression among female neurology residents, although gender did not always remain an independent predictor after multivariable adjustment^7,10^. Furthermore, studies conducted during periods of heightened occupational stress, such as the COVID-19 pandemic, reported significantly higher rates of depression among female physicians and healthcare workers^13,15^. From a biological perspective, evidence suggests that females may exhibit greater hypothalamic–pituitary–adrenal axis reactivity to chronic stress, increasing vulnerability to depression under sustained occupational demands^1,2^, which may partially explain the higher odds observed in the adjusted model.

In contrast, several studies suggest that gender alone may not be a robust independent predictor of depressive symptoms among physicians once occupational and psychosocial factors are considered. Chen et al. (2022) found no significant association between gender and depression among emergency physicians after adjusting for workload, burnout, and sleep disturbances^12^, indicating that observed gender differences may be confounded by work-related stressors. Similarly, Tomioka et al. (2011) reported that long working hours and occupational stress were stronger predictors of depression than gender among physicians^16^. Evidence also indicates that male physicians may underreport depressive symptoms or be less likely to seek mental health care due to stigma, potentially leading to an underestimation of depression prevalence in men^5^. Additionally, a systematic review among surgeons by Rogers and McCulloch (2023) found inconsistent gender differences in depression and anxiety, suggesting that specialty-specific cultures and stressors may attenuate gender effects^11^. Studies from diverse cultural settings have also reported mixed or non-significant gender differences in physician depression^8,14^, underscoring the influence of contextual and regional factors.

### Job title, years of experience and working hours

The study further identified a notable association between depressive symptom severity and both years of clinical experience and job title. Among neurologists with fewer than three years of professional experience, 53.1% exhibited moderate to severe depressive symptoms. In contrast, only 19.4% of those with more than ten years of experience reported similar symptom severity. Regarding professional rank, residents reported the highest mean depression score (M = 11.4), followed by those categorized as “Other” (M = 12.4) and registrars (M = 9.2). In comparison, consultants and professors reported significantly lower scores. These trends are consistent with prior research, including a 2015 systematic review which evaluated 54 studies that assessed depressive symptoms using mixture of clinical interviews, PHQ-9 scores and 2-item PRIME-MD, they found that depressive symptoms were significantly more prevalent among medical trainees, particularly following the onset of residency training with median absolute increase of 15.8%^6^. Moreover, a study conducted on 65 neurology residents at Universitas Indonesia (Putri et al.) assessed depression using the BDI-II tool found that the job title or level of training was the most significant factor^10^. Junior-level residents (those in the early years of their specialty training) were significantly more likely to experience depression compared to senior residents (p = 0.044)^10^. Overall Prevalence: 18.4% of the neurology residents had depression (9.2% mild and 9.2% moderate)^10^. No residents in this specific study were found to have severe depression^10^.

It’s worth mentioning that a meta-analysis by wen et al. (2024) that was done on 10 studies that combined a total of 2,389 orthopedic residents for depression analysis described a significant correlation between Job title (PGY level) and depression. The meta-analysis found that junior residents (specifically PGY-2 and PGY-3) often had higher rates of depressive symptoms compared to research residents or very senior residents.

The consensus of results between our findings and the cited research regarding the inverse relationship between seniority and depression can be explained using well-established occupational stress models. According to the Job Demand–Control (JDC) Model (Karasek, 1979)^17^, junior residents are exposed to high psychological demands (long hours, clinical responsibility, frequent evaluations) while having limited control over decision-making. This combination places them in a “high strain” work environment, which is strongly associated with psychological distress and depression^17^.

In addition, the Transactional Theory of Stress (Lazarus & Folkman, 1984)^18^ helps explain why junior physicians are particularly vulnerable. Less experienced clinicians are more likely to interpret complex clinical situations as threats rather than challenges, due to limited clinical confidence and coping experience. This threat-based appraisal increases emotional stress and depressive symptoms. As experience accumulates, physicians are more likely to reframe similar situations as manageable challenges, reducing emotional burden^18^.

Regarding working hours, statistical analysis revealed a moderate and statistically significant positive correlation between weekly working hours and PHQ-9 depression scores among neurologists, indicating that longer work schedules were associated with depressive symptoms particularly those exceeding 72 h. This was contradictory in the study by Putri et al. (2021). Where hours worked per week were not significantly associated with the occurrence of depression^10^. In fact, that it was more associated with occupational stressors and overcommitment regardless of the number of working hours^16^. It is also noteworthy that the meta-analysis by Wen et al. (2024), identified a significant association between extended working hours and depression. The analysis showed that residents working more than 60 h per week, as well as those spending over 9 h per day at work, exhibited greater depression severity^19^.

The discrepancy between our findings and those of Putri et al. (2021) may be understood by considering both psychosocial and biological mechanisms. The Effort–Reward Imbalance (ERI) Model (Siegrist, 1996)^20^ proposes that long working hours contribute to depression primarily when high effort is not balanced by adequate rewards, such as professional recognition, support, or career advancement^20^. In the Putri et al. cohort, depression appeared to be driven more by workplace stressors and overcommitment than by working hours alone. While the biological aspect is more explained through the Neurobiological evidence from Yoo et al. (2007)^21^ demonstrating that sleep deprivation impairs functional connectivity between the prefrontal cortex and the amygdala, reducing emotional regulation and increasing vulnerability to negative effect^22^. This mechanism provides a plausible explanation for why extremely long working hours show a stronger association with depressive symptoms in our cohort.

### History of depression

A strong association was also observed between depression severity and self-reported history of depression. Among participants with a prior diagnosis of depression, 82.4% (n = 28) reported moderate to severe symptoms, whereas only 17.6% (n = 6) reported none or mild symptoms. Conversely, among those without a documented history of depression, 62.5% (n = 65) reported none or mild symptoms, while 37.5% (n = 39) experienced moderate to severe symptoms. In addition, multivariate analysis revealed that a prior diagnosis of depression before residency was strongly associated with current depression (PHQ-9 ≥ 10), with substantially higher odds among those reporting a prior diagnosis (aOR 6.71, 95% CI 1.65–27.32; p≈0.008). These findings are supported by a recent systemic review by Rogers and McCulloch (2023) that included 31 studies with a combined total of 11.399 surgeons where they assessed depression using various tools most commonly the GHQ-12, PHQ-9 and HADS. The review identifies a prior history of mental health conditions as a significant predictor for current depressive symptoms. Surgeons with a history of depression were at a much higher risk of recurrence, especially when facing high-stress events like surgical complications or medical-legal issues^11^.

The similarities between our results and Rogers and McCulloch (2023) can be explained by Kindling Theory (Post, 1992)^21^, which posits that initial depressive episodes “prime” the brain, lowering the threshold for future stressors to trigger recurrence^21^. In high-pressure specialties like neurology and surgery, this neurobiological sensitization makes those with a history significantly more vulnerable to routine occupational stress. Our study did report higher levels of depression for those without a documented history of depression compared to Rogers and McCulloch (2023), This may reflect specialty-specific demands or a more sensitive screening tool (PHQ-9) compared to the varied tools (GHQ-12, HADS) used in the surgical meta-analysis.

Considering these findings, future research should adopt a more comprehensive approach to investigating and addressing mental health among neurologists, particularly within the hospital setting. Second, further studies with larger sample sizes are needed to better evaluate the relationships between depression and associated demographic, occupational, and clinical factors.

## Limitation

This study has several limitations. It was conducted on a small number of neurologists and was limited to the Cairo and Giza governorates, which may affect the generalizability of the findings. Important variables such as marital status, type of institution (public vs. private), and underlying medical condition, which could influence the risk of depression, were not assessed. Although all eligible neurologists working in the eight selected centers during the study period were approached and invited to participate, an exact denominator of the total number of neurologists could not be precisely determined due to variations in staffing numbers and clinician availability, particularly in university hospitals. Non-response occurred due to refusal or unavailability at the time of data collection, which may introduce non-response bias. Additionally, there is a scarcity of research specifically examining depression among neurologists. Most of the studies focus on other specialties, such as orthopedic surgeons or emergency physicians. While these comparisons provide useful context, they may not fully capture the unique demands, stressors, and work patterns of neurology practice, so caution is needed when interpreting differences in prevalence and severity of depressive symptoms across specialties.

## Conclusion

This study reveals a high prevalence of depressive symptoms among Egyptian neurologists, with nearly half experiencing moderate to severe levels of depression. The burden was markedly greater among those with a prior history of depression, neurologists starting early in their career, and individuals working excessive hours. These findings suggest that both mental health history and workplace factors, such as workload and professional seniority, play a critical role in shaping mental well-being. Addressing mental health among neurologists requires a multifaceted approach, that should include routine psychological assessment, support for neurologists early in their career, and systematic approach to reduce burnout and workload. Investing in the mental health of neurologists is not only vital for their well-being but also for the quality of care they provide.

## Recommendations

Preforming a wide base national study including all governates in Egypt and using a more detailed questionnaire other than PHQ-9.

## Data Availability

The data that support the findings of this study are not openly available due to reasons of sensitivity and are available from the corresponding author upon reasonable request.

## Abbreviations

BDNF: Brain-derived neurotrophic factor
HPA: Hypothalamic–pituitary–adrenal axis
aOR: Adjusted odds ratio
OR: Odds ratio
PHQ-9: Patient health questionnaire-9
PRIME-MD: Primary care evaluation of mental disorders
GHQ-12: 12-Item general health questionnaire
HADS: Hospital anxiety and depression scale
ERI: Effort-reward imbalance
JDC: Job-demand control
BDI-II: Beck depression inventory 2nd edition
PGY: Post-graduate year
